# Deficient recovery response and adaptive feedback potential in dynamic gait stability in unilateral peripheral vestibular disorder patients

**DOI:** 10.14814/phy2.12222

**Published:** 2014-11-27

**Authors:** Christopher McCrum, Katrin Eysel‐Gosepath, Gaspar Epro, Kenneth Meijer, Hans H. C. M. Savelberg, Gert‐Peter Brüggemann, Kiros Karamanidis

**Affiliations:** 1 Human Movement Science, NUTRIM, School for Nutrition, Toxicology and Metabolism, Maastricht University, Maastricht, The Netherlands; 2 Department of Otolaryngology, Head and Neck Surgery, Heinrich Heine University of Düsseldorf, Düsseldorf, Germany; 3 Institute of Biomechanics and Orthopaedics, German Sport University Cologne, Cologne, Germany; 4 Institute of Movement and Sport Gerontology, German Sport University Cologne, Cologne, Germany; 5 Cologne Center for Musculoskeletal Biomechanics, Medical Faculty, University of Cologne, Cologne, Germany; 6 Department of Mathematics and Technology, University of Applied Sciences Koblenz, RheinAhrCampus Remagen, Remagen, Germany

**Keywords:** Fall risk, gait adaptation, margin of stability, vestibular dysfunction

## Abstract

Unilateral peripheral vestibular disorder (UPVD) causes deficient locomotor responses to novel environments due to a lack of accurate vestibular sensory information, increasing fall risk. This study aimed to examine recovery response (stability recovery actions) and adaptive feedback potential in dynamic stability of UPVD‐patients and healthy control subjects during perturbed walking. 17 UPVD‐patients (>6 months since onset) and 17 matched healthy control participants walked on a treadmill and were subjected to eight unexpected perturbations during the swing phase of the right leg. For each perturbation, the margin of stability (MS; state of body's centre of mass in relation to the base of support), was determined at touchdown of the perturbed leg and during the following six recovery steps. The first perturbation caused a reduced MS at touchdown for the perturbed leg compared to baseline, indicating an unstable position, with controls requiring five recovery steps to return to MS baseline and UPVD‐patients not returning to baseline level within the analyzed six recovery steps. By the eighth perturbation, control subjects needed two steps, and UPVD‐patients required three recovery steps, both thereby improving their recovery response with practice. However, MS at touchdown of the perturbed leg increased only for the controls after repeated perturbations, indicating adaptive feedback‐driven locomotor improvements for the controls, but not for the UPVD‐patients. We concluded that UPVD‐patients have a diminished ability to control dynamic gait stability during unexpected perturbations, increasing their fall risk, and that vestibular dysfunction may inhibit the neuromotor system adapting the reactive motor response to perturbations.

## Abstract (precis)

Dynamic gait stability was assessed in 17 unilateral peripheral vestibular disorder patients and 17 healthy controls during perturbed walking on the treadmill. Unilateral peripheral vestibular disorder was associated with deficient locomotor recovery responses to the perturbation task and a lack of adaptation feedback potential following repetition of the perturbation task compared with controls.

## Introduction

Human biped locomotion is a mechanically intricate motor task due to the need to produce effective and safe gait patterns during daily life. Such complexity is required to cope with changing environmental demands such as steps, slopes, or uneven terrain. To safely negotiate such environmental conditions, maintain balance, and avoid trips and falls while walking, effective postural adjustments and locomotor adaptations are required. Motor adaptations to environmental changes can be immediate reactive responses that rely on ongoing afferent feedback information, or feedforward predictive adjustments (Marigold and Patla 2002; Pai et al. 2003; Pavol et al. 2004; Bhatt et al. 2006; Lam et al. 2006). It is suggested that central pattern generators may be responsible for spinal structures controlling basic gait patterns during reactive responses, with the cerebellum controlling feedforward, predictive adjustments (Morton and Bastian 2006).

A number of recent studies have demonstrated an improvement in the effectiveness of such feedforward adjustments (i.e., adjustments made in preparation for a given perturbation based on prior knowledge and experience of the constraint) and/or reactive responses (i.e., adjustments made as a direct response following a perturbation) after repeated practice of different task constraints (Marigold and Patla 2002; Pai et al. 2003; Pavol et al. 2004; Bhatt et al. 2006; Bierbaum et al. 2010, 2011). For example, Bierbaum et al. (2010, 2011) reported favorable adaptations in feedforward adjustments and reactive responses in dynamic gait stability after repeated exposure to changes in surface compliance while walking in older and younger adults. The adaptations were observed as increases in margin of stability indicating a reduced fall risk (Bierbaum et al. 2010, 2011). The margin of stability (Hof et al. 2005) quantifies the stability of the body configuration using the difference between the base of support and the extrapolated center of mass (calculated using the position and velocity of the center of mass). Negative margin of stability values indicate an unstable body configuration, whereas positive margin of stability values indicate a stable body configuration (i.e., no further motor actions are needed to preserve postural stability). The degree to which the reactive response can improve, known as adaptive feedback potential, describes the reactive, feedback‐driven adaptations to unexpected perturbations that occur over time (Pavol et al. 2004; Bierbaum et al. 2011). Bierbaum et al. (2011) analyzed margin of stability at touchdown of the first recovery step which was expected to reveal a predominantly reactive‐driven motor response, due to the sudden and unexpected nature of the perturbation. As knowledge and experience gathered over time for specific task constraints can positively influence recovery performance, the integration of accurate sensory feedback information during the perturbations may be significant for the success of such movement corrections and adaptations during locomotion.

Sensory feedback is important for successful and safe locomotion, as it informs the central nervous system about the actual state of the musculoskeletal system and the environmental conditions (Wolpert et al. 1995; Sainburg et al. 1999; Rossignol et al. 2006). In particular, the vestibular system plays an important role in gait, providing information regarding the position, velocity and direction of the head in space (Kennedy et al. 2003; Rossignol et al. 2006) and contributing to lower limb control (Bent et al. 2005). Additionally, gait trajectory is reported to be negatively affected when vestibular sensory input is disturbed, showing greater variability during artificial vestibular perturbations (Kennedy et al. 2003). Therefore, deficiencies in stability control during gait could be expected if the vestibular system is dysfunctional.

A specific group that experiences vestibular dysfunction is unilateral peripheral vestibular disorder (UPVD) patients. UPVD includes disorders of the inner ear on one side such as vestibular neuritis (Hillier and McDonnell 2011). UPVD is associated with imbalance and falls (Neuhauser et al. 2005; Homann et al. 2013) and is the leading cause of dizziness (Lüscher et al. 2014). It has been shown that UPVD patients struggle to maintain consistent arm actions during trunk movement without visual information (Raptis et al. 2007) and demonstrate deficiencies during goal‐directed reaching tasks while both seated and standing (Borello‐France et al. 1999). As upper body motor tasks during standing and sitting are negatively affected by UPVD, dynamic stability control during perturbed walking may also be disrupted by UPVD. Consequently, adaptations of such motor behaviors over time may also be diminished. Some level of motor response and adaptation may be possible through spinal structures driving reactive responses, as has been reported in human infants (Lam et al. 2003; Pang et al. 2003). There is evidence to suggest that vestibular and somatosensory information interact at the spinal level during reactive postural control (Horak et al. 2001), but it has not, to our knowledge, been investigated if recovery performance and reactive locomotor responses during perturbed walking are affected by UPVD.

Therefore, this study aimed to examine the recovery responses and adaptive feedback potential in dynamic stability of UPVD patients and matched healthy control subjects during perturbed treadmill walking, by repeatedly applying unilateral resistance unexpectedly to the swing phase of the right leg. In order to predominantly examine the reactive response of the participants, we determined the recovery stepping behavior of the participants at touchdown of the perturbed leg. We defined the reactive response using the above described method as the perturbation was unexpected with no prior warning given, and the duration between onset of perturbation and touchdown was short (also see Süptitz et al. 2013). It was hypothesized that recovery stepping behavior would be less effective and margin of stability at touchdown of the perturbed leg would demonstrate less improvement with practice in UPVD patients compared to matched healthy controls after repeated exposure to the perturbation, indicating a diminished adaptive feedback potential in dynamic stability during perturbed walking in UPVD patients. Such findings could be relevant for fall risk reduction interventions in UPVD patients and could enhance our understanding of the role of the vestibular system in dynamic stability control during locomotion.

## Materials and Methods

### Subjects

Seventeen adult patients diagnosed with UPVD recruited from a medical clinic (10 females and 7 males; age: 49 years (SD9); body height: 171.4 cm (SD7.3); body mass: 73.8 kg (SD14.1)) and 17 matched healthy adults [10 females and 7 males; 51 years (SD8); body height: 172.5 cm (SD8.2); body mass: 75.1 kg (SD15.2)] participated in this study. Healthy adults were selected as control subjects based on gender, age, anthropometric measures, and physical activity level (frequency of participation in physical activity per week, determined by questionnaire), to create matched pairs with the UPVD patients. Patients with diagnosed vestibular neuritis, that were at least 6 months since onset, were recruited. The patients were experiencing rotational vertigo and eight suffered from feelings of instability and unsteadiness. Four patients had fallen in the previous 6 months. Patients were assessed for inclusion and had their diagnoses confirmed by a specialist otolaryngologist during a clinical examination that included examining for spontaneous and induced vestibular nystagmus, bithermal caloric tests under videostagmography, head impulse tests, rotating chair tests, and the examination of balance and coordination using Romberg and Unterberger tests. Fifteen patients had a right side deficit and two patients had a deficit on the left side. The subjects of the healthy control group were also medically screened by the same otolaryngologist using identical tests to confirm that they did not have UPVD. Patients were excluded if their time since UPVD onset was less than 6 months or if their diagnosis was not confirmed. Further inclusion criteria for the patients and the control subjects were that they did not participate in sport or physical exercise more than once per week and had no other health problems, including cardiovascular disease, musculoskeletal injuries, or any other locomotor dysfunction. The study was approved by the university's ethical board, the procedures of the study were explained to the participants, and written informed consent was obtained prior to the testing in accordance with the Declaration of Helsinki.

### Experimental setup

All subjects walked on a motor‐driven treadmill (pulsar 4.0, h/p/cosmos; Nussdorf‐Traunstein, Germany) with a belt speed of 1.4 m·s^−1^. Familiarization with the treadmill was carried out at least once for each subject 4–7 days prior to data collection. To achieve natural movement patterns, all participants were asked to wear their own regular sports shoes. Subjects always wore a safety harness connected to an overhead track. No instructions were given to the subjects regarding gaze fixation.

**Figure 1.**
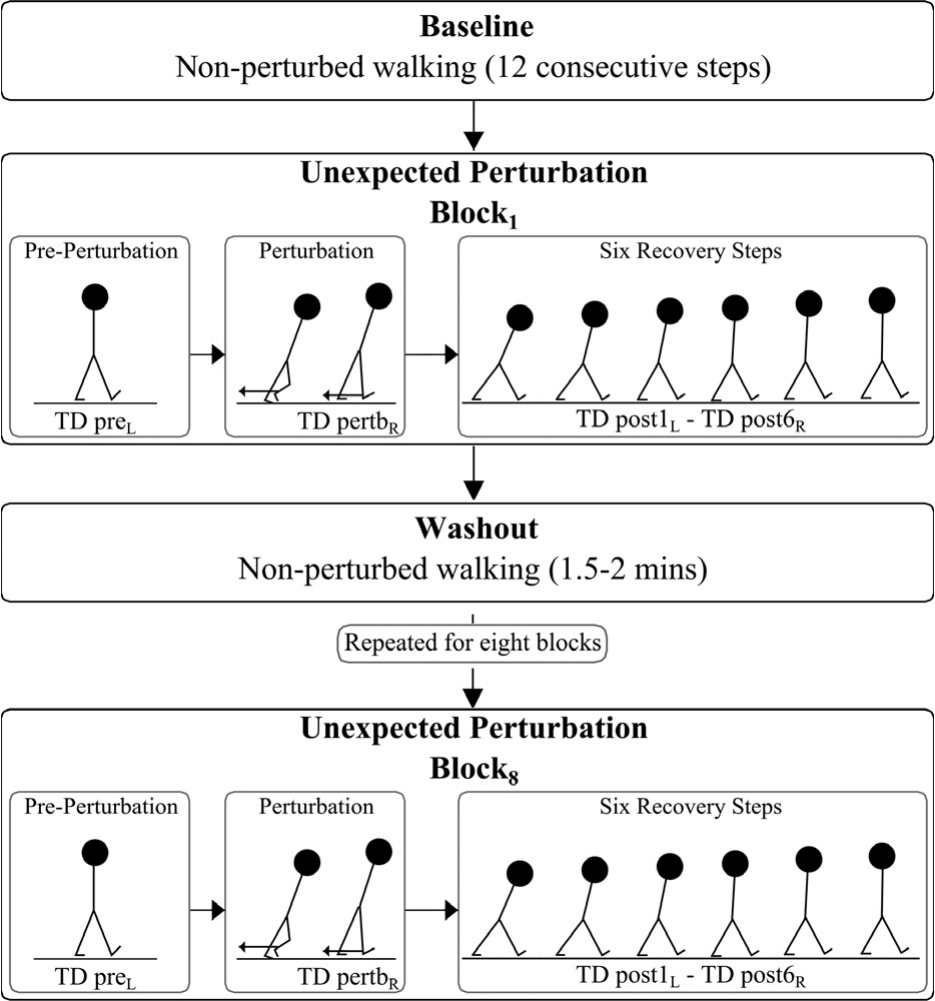
Experimental protocol of the gait perturbation task: The protocol began with a baseline period (nonperturbed walking), followed by eight unexpected perturbation blocks separated by washout periods. Baseline was defined by averaging 12 consecutive steps of nonperturbed walking. Gait perturbation was accomplished by using an unexpected (subjects were not warned) application of resistance using a custom built electronically driven magnet system during the swing phase of the right leg. For each perturbation block, dynamic stability parameters were examined at touchdown (TD) of the contralateral step of the left leg prior to perturbation (pre_L_), TD of the perturbed step (pertb_R_) and TD of the six recovery steps following each perturbation (post1_L_–post6_R_). Washout periods between blocks (typically 1.5–2 min) were given, so that postural adjustments made due to the applied resistance dissipated prior to the next block.

The exact gait perturbation task and protocol has been described previously by Süptitz et al. (2013). Briefly, on the day of the measurements, the gait protocol started with five minutes of walking at 1.4 m·s^−1^ to allow the subjects to get accustomed once more to the treadmill and gait velocity. Following that, a Teflon rope was connected with Velcro straps to the subjects' right leg above the ankle joint. The rope was connected to a custom built device which was used to apply and remove unexpectedly a unilateral resistance of 2.1 kg during the swing phase of the right leg using an electronically driven magnet system (see Süptitz et al. 2013). Participants then walked at 1.4 m·s^−1^for 3–4 min without perturbation and a baseline period (nonperturbed walking) of about 20 sec of walking was recorded at the end of this period (Fig. 1). Following the baseline period, the resistance was turned on for the entire duration of the swing phase of the right leg for one step and was subsequently turned off (Süptitz et al. 2013). This one‐time application of resistance was unexpected for all participants. The instant of foot touchdown at the contralateral step of the left leg prior to perturbation (pre_L_), the perturbed step (pertb_R_) and the six recovery steps following each perturbation (post1_L_–post6_R_), collectively defined as unexpected perturbation block one (block_1_), were of interest for the analysis of the dynamic stability parameters (see Fig. 1; exact parameters are described below). These specific gait events were analyzed in order to determine if any alterations in gait occurred preperturbation due to preparation behavior or anxiety (pre_L_) and to examine the response behavior of the participants during (pertb_R_) and postperturbation (post1_L_–post6_R_). The above described unexpected perturbation application and analysis procedure was repeated for a total of eight unexpected perturbation blocks (block_1_ to block_8_; see Fig. 1) within a period of approximately 25 min of walking. Between perturbation blocks sufficient time (typically 1.5–2 min) was given as a “washout” period, so that postural adjustments made due to the applied resistance dissipated (Fig. 1). The washout was controlled for each individual using a live observation of the anteroposterior displacement of the toe markers while walking, using a motion capture system (Vicon Motion Systems, Oxford, UK), to determine the step length compared to baseline values (nonperturbed gait). In cases where clear differences (more than 10%) of the anteroposterior displacement of the toe markers compared with baseline existed longer than the typical 1.5–2 min washout period, the washout period was extended as long as was necessary for the values to return to baseline. However, this was not required for any subjects, with most returning to baseline within 1 min. Subjects were never warned about the application or removal of the resistance.

### Dynamic stability analysis during walking

The method used to analyze dynamic stability during treadmill walking has been described in detail in two previous studies (Süptitz et al. 2012, 2013). Briefly, to track a twelve‐segment, full body kinematic model (left and right foot, left and right lower leg, left and right thigh, trunk, left and right lower arms, left and right upper arms, head) and to examine gait kinematics, 26 retro reflective markers (radius 16 mm) were attached to anatomical landmarks on the skin and the 3D coordinates of the markers were recorded by the Vicon Nexus motion capture system. The motion capture system was comprised of eight infrared cameras operating at 120 Hz and the 3D coordinates of the markers were smoothed using a Woltring filter routine with a mean squared error of five (Woltring 1986). Segmental masses and their location were calculated based on the data reported by Dempster et al. (1959).

**Figure 2.**
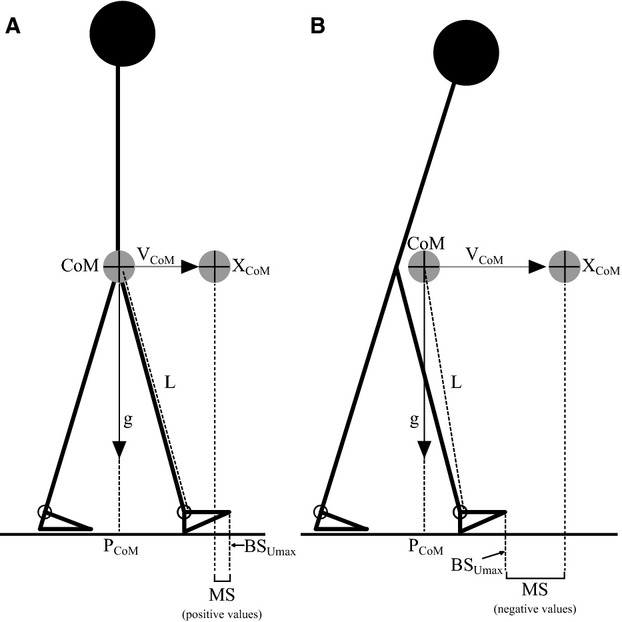
Schematic illustration of the inverted pendulum model during locomotion (Hof et al. 2005). P_C__oM_ represents the horizontal (anteroposterior) component of the projection of the center of mass (CoM) to the ground, V_C__oM_ represents the horizontal velocity of the CoM (anteroposterior), g is acceleration due to gravity and L is the pendulum length (i.e., distance between the CoM and the centre of the ankle joint in the sagittal plane). Margin of stability (MS) in the anterior direction is the instantaneous difference between the anterior boundary of the base of support (BS_U__max_) and the extrapolated center of mass (X_C__oM_)_._ Positive MS values (A) indicate a stable body configuration (during unperturbed walking this would mean that additional motor actions would be required to continue walking, such as leading leg hip extensor and/or trailing leg ankle plantarflexor action), whereas negative margin of stability values (B) indicate an unstable body configuration (i.e., subjects must make additional motor actions to preserve stability and to avoid a fall, e.g., by stepping).

The margin of stability in the anteroposterior direction during walking was determined using the extrapolated centre of mass concept (Hof et al. 2005; see also Fig. 2). Margin of stability was calculated as the difference between the anterior boundary of the base of support (horizontal component of the projection of the toe from the corresponding limb to the ground) and the extrapolated center of mass (Fig. 2). Extrapolated center of mass (*X*_CoM_) was defined as follows: 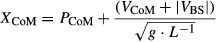
where *P*_CoM_ represents the horizontal (anteroposterior) component of the projection of the CoM to the ground, *V*_CoM_ represents the horizontal velocity of the CoM (anteroposterior), *V*_BS_ represents the horizontal (anteroposterior) velocity of the anterior boundary of the base of support (calculated by the average velocity of the forefoot markers during the stance phase, approximately equal to the speed of the treadmill belt), g is acceleration due to gravity, and L is the distance between the CoM and the centre of the ankle joint in the sagittal plane (Fig. 2).

The base of support was defined as the distance between the anterior boundaries of the leading and trailing foot at touchdown, using the distance between the vertical projections of the respective toe markers and the extrapolated center of mass at touchdown was calculated with reference to the anterior boundary of the base of support (leading leg; Fig. 2). The touchdown was determined using the acceleration of the tibia, measured by two‐dimensional accelerometers (±50 g; ADXL250; Analog Devices, Norwood, MA, USA) attached with tape to the midpoint of the tibias (Süptitz et al. 2012). Baseline values for margin of stability, extrapolated center of mass and base of support were calculated by averaging 12 consecutive steps (left and right; base, see Fig. 1) of nonperturbed walking for each subject. For the analysis of the locomotor response to the perturbation and the postural corrections before and after repeated practice of the perturbation task, the perturbed right leg and at the six recovery steps of the first and final perturbation blocks (perturbation block_1_ and perturbation block_8_) were considered in the statistics.

The extent of the adaptation of the reactive response due to the adaptive feedback potential of the participants was calculated using margin of stability at touchdown as follows: 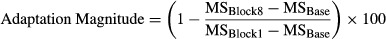
where MS_Block1_ and MS_Block8_ are margin of stability at touchdown of the perturbed leg of the first and eighth blocks (pertb_R_ in block_1_ and pertb_R_ in block_8_), respectively, with margin of stability baseline represented by MS_Base_. Four patients with UPVD were unable to cope with the gait task, and were only able to prevent a fall after the sudden perturbation by grasping the treadmill handrails. Those four patients and their matched healthy control subjects were excluded from the analysis and, hence, only 13 patients and 13 control subjects were included in the statistics.

### Statistics

A three‐way repeated measures analysis of variance (ANOVA), with subject group (UPVD patients and healthy controls), gait event (base, pertb_R_, post1_L_–post6_R_; dependent variable) and perturbation block (block_1_ and block_8_; dependent variable) as factors was used to determine differences in the margin of stability, extrapolated center of mass and base of support and, hence, to examine the recovery response of the participants before and after repeated practice of the unexpected perturbation task. For the analysis of the adaptive feedback potential of the UPVD patients and the controls, the perturbed right leg (pertb_R_) of all eight examined perturbation blocks (block_1_ to block_8_) were considered. A two‐way repeated measures ANOVA, with subject group (UPVD patients and healthy controls) and perturbation block (block_1_ to block_8_) as factors was used in order to examine and identify any subject group or perturbation block related differences in margin of stability and postural corrections during the course of the perturbations. Possible locomotor adjustments of the participants prior to each perturbation were checked by examining the margin of stability, extrapolated center of mass and base of support for each step preperturbation (pre_L_ in block_1_ to block_8_) in relation to baseline by using a two‐way ANOVA with subject group and perturbation block as factors. For each significant result, we applied simple contrasts to further investigate whether the outcome measures at certain gait events (pre_L_, pertb_R_, post1_L_–post6_R_) differed from baseline or whether differences between subject groups or perturbation blocks existed. The adaptation magnitude of the UPVD patients and healthy controls was checked for differences using an independent samples t‐test. The level of significance for all tests was set at *α *= 0.05. Before applying the statistical analyses, the distribution normality of our results for each variable was checked using the Kolmogorov–Smirnov Test, which revealed normal distributions (*P* values > 0.05). Statistical analyses were carried out with STATISTICA 7.1 (StatSoft Inc., Tulsa, OK, USA). All results are presented as mean and standard deviation (mean and SD).

## Results

### Clinical examination

All examined participants of the UPVD patient group showed clear balance and coordination deficits (16 failed the Unterberger test, 12 failed the straight line walking with eyes closed test, nine showed deficits in the head impulse tests) and six patients had forms of nystagmus (one case of spontaneous nystagmus, five showed positive results for head‐shaking nystagmus). The clinical examinations of the control group confirmed that all but one subject had any form of nystagmus, and no clear balance and coordination problems were identified. Only one control subject failed the Unterberger test, but this may be attributed to a lack of concentration, and as the subject showed no other indications or symptoms of UPVD, the subject was not excluded from the study.

### Recovery locomotor behavior before and after experience of eight perturbations

**Figure 3.**
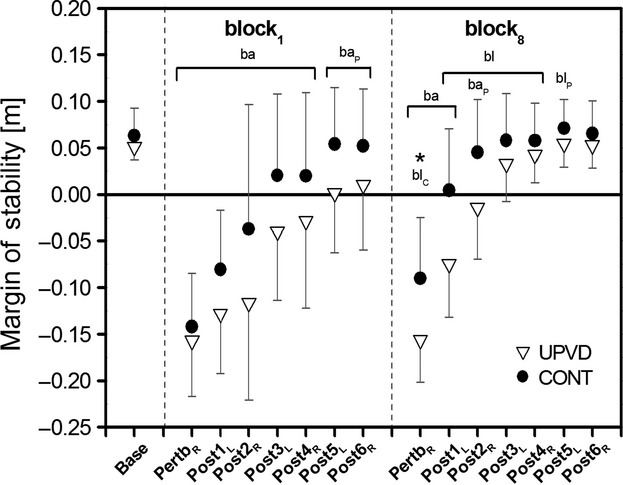
Margin of stability at touchdown (TD) during nonperturbed walking (base: average values of 12 consecutive steps), at TD of the perturbed leg (pertb_R_), and at TD of the following six consecutive steps to an unexpected perturbation (post1_L_–post6_R_) for unilateral peripheral vestibular disorder (UPVD;* n *=**13) patients and matched healthy controls (CONT;* n *=**13) during the first (block_1_) and the final (block_8_) unexpected perturbation during treadmill walking (set speed of 1.4 m·s^−1^; mean and SD). Negative margin of stability values indicate an unstable body configuration (i.e., subjects must make additional motor actions to preserve stability and to avoid a fall, e.g., by stepping), whereas positive margin of stability values indicate a stable body configuration (i.e., no additional motor actions are needed to preserve stability). ba: Statistically significant difference to base for both subjects groups (*P* < 0.05). ba_P_: Statistically significant difference to base for the UPVD group (*P* < 0.05). bl: Statistically significant difference to block_1_ for both subjects groups (*P* < 0.05). bl_C_: Statistically significant difference to block_1_ for the control group (*P* < 0.05). bl_P_: Statistically significant difference to block_1_ for the UPVD group (*P* < 0.05). *Statistically significant difference between the UPVD and control groups (*P* < 0.05).

**Figure 4.**
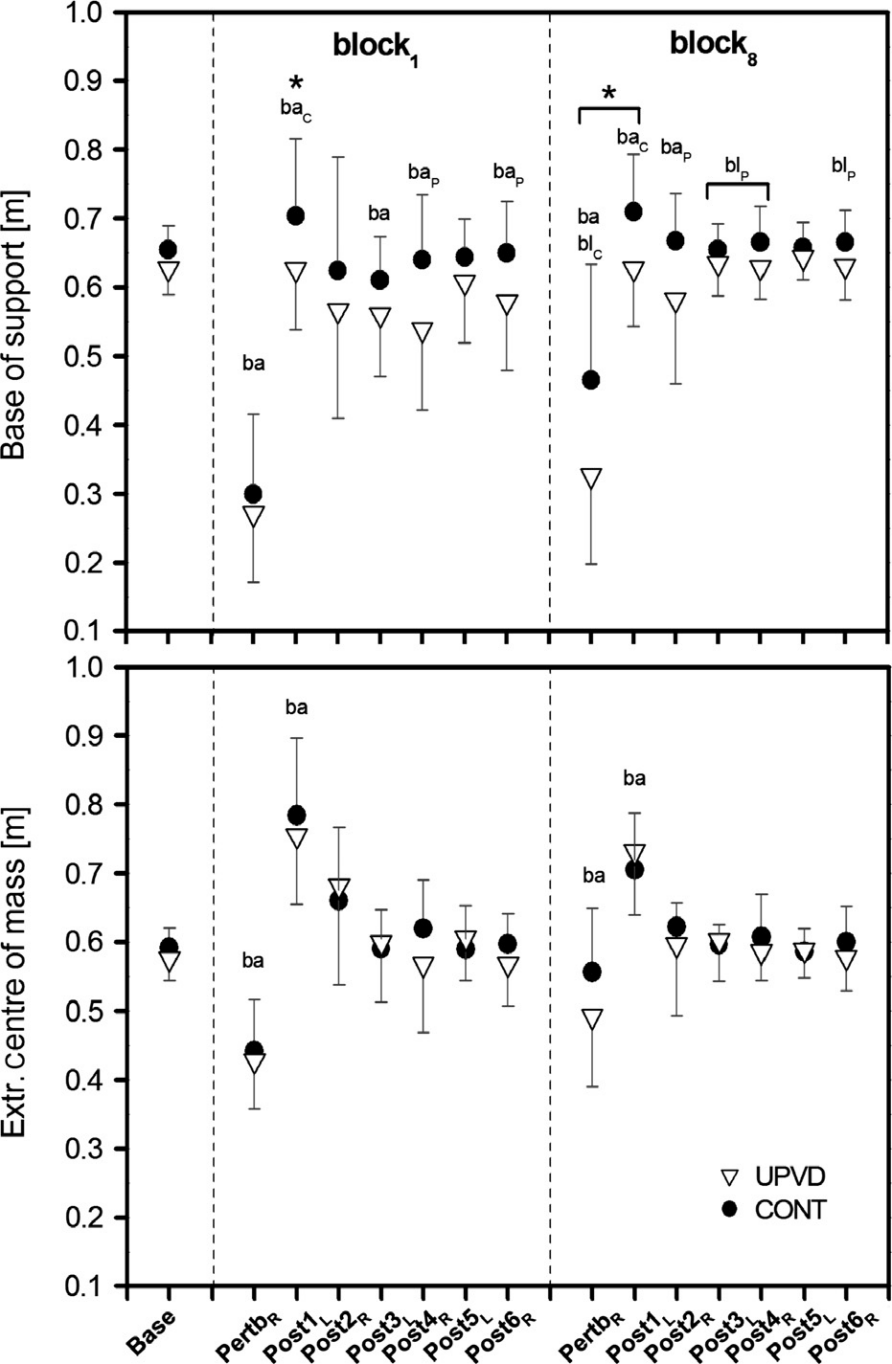
Base of support and extrapolated centre of mass at touchdown (TD) during nonperturbed walking (base: average values of 12 consecutive steps), at TD of the perturbed leg (pertb_R_), and at TD of the following six consecutive steps to an unexpected perturbation (post1_L_–post6_R_) for unilateral peripheral vestibular disorder (UPVD;* n *=**13) patients and matched healthy controls (CONT;* n *=**13) during the first (block_1_) and the final (block_8_) unexpected perturbation during treadmill walking (set speed of 1.4 m·s^−1^; mean and SD). In order to maintain a stable body configuration (i.e., positive margin of stability) the base of support must exceed the extrapolated center of mass. Please note that for the base of support there were tendencies (*P* < 0.1) for differences from baseline for the UPVD patients in block_1_ for post2_R_ and post5_L_. ba: Statistically significant difference to base for both subjects groups (*P* < 0.05). ba_C_: Statistically significant difference to base for the control group (*P* < 0.05). ba_P_: Statistically significant difference to base for the UPVD group (*P* < 0.05). bl_C_: Statistically significant difference to block_1_ for the control group (*P* < 0.05). bl_P_: Statistically significant difference to block_1_ for the UPVD group (*P* < 0.05). *Statistically significant difference between the UPVD and control groups (*P* < 0.05).

The analysis of dynamic stability control during walking (nonperturbed gait and the first and last unexpected perturbation blocks) revealed a significant (*P* < 0.05) perturbation block × gait event × subject group interaction for base of support and margin of stability, meaning the effect of subject group on dynamic stability control was gait event and perturbation block specific. A simple contrast test revealed no significant differences in base of support and margin of stability at touchdown between UPVD patients and controls during nonperturbed walking (base; Figs. 3 and 4).

The application of unexpected unilateral resistance to the right leg while walking caused the margin of stability to significantly decrease (*P* < 0.05) at touchdown of the perturbed right leg (pertb_R_) for both subject groups and for both perturbation block_1_ and perturbation block_8_, with no significant differences between subject groups in the first perturbation block (Fig. 3). However, the control group showed a significant (*P* < 0.05) increase in margin of stability at touchdown of the perturbed leg in perturbation block_8_ in comparison to block_1_, resulting in significantly lower margin of stability values for the UPVD patients compared to the control group in perturbation block_8_ (*P* < 0.05; Fig. 3). The extrapolated center of mass showed a significant gait event × perturbation block interaction (*P* < 0.05). Compared to baseline, the base of support and the extrapolated center of mass were significantly (*P* < 0.05) lower for both groups and both unexpected perturbation blocks at touchdown of the perturbed right leg (Fig. 4). However, the control group showed a significant (*P* < 0.05) increase in base of support at touchdown of the perturbed leg in perturbation block_8_ in comparison to block_1_, whereas no significant differences between blocks were found for the UPVD patients (Fig. 4).

At touchdown of the first step following the first and final unexpected perturbations (post1_L_ in block_1_ and block_8_), the base of support was significantly (*P* < 0.05) higher compared to baseline walking for the control group, whereas no significant differences were found for the UPVD patients (Fig. 4). Compared to the control group, the UPVD patients showed a lower base of support at touchdown of the first step following the perturbation independent of perturbation block (*P* < 0.05; Fig. 4). During the following five consecutive steps (post2_R_–post6_R_) in perturbation block_1_, the UPVD patients showed lower base of support values compared to baseline (*P* values were not <0.05 for all steps; Fig. 4) and did not reach a steady state within the analyzed six steps following the unexpected perturbation (post1_L_–post6_R_). However, in the final perturbation block (block_8_), the UPVD patients showed significantly (*P* < 0.05) higher base of support values in comparison to perturbation block_1_ (Fig. 4), and reached baseline level base of support values within the third step following the perturbation (post3_L_). The extrapolated center of mass significantly increased (*P* < 0.05) above baseline for both groups and both blocks at post1_L_, but showed no significant subject group effect or subject group interaction (subject group × gait event, subject group × block, subject group × block × gait event; Fig. 4).

As a consequence of the above findings, the UPVD patient group demonstrated significantly (*P* < 0.05) lower margin of stability values for the steps following the perturbation compared to baseline walking (for perturbation block_1_: post1_L_–post6_R_; block_8_: post1_L_–post2_R_) with a significant increase in margin of stability from perturbation block_1_ to perturbation block_8_ (post1_L_–post5_L_; *P* < 0.05). Hence, for perturbation block_1_ and perturbation block_8_, the UPVD patients required more than six and three recovery steps, respectively, to return to the margin of stability baseline level (*P* < 0.05; Fig. 3). Comparatively, the healthy control subjects needed for perturbation block_1_ and perturbation block_8_ five and two recovery steps to return to margin of stability baseline, respectively, (*P* < 0.05; Fig. 3), with a significant (*P* < 0.05) increase in margin of stability from block_1_ to block_8_ for the first four steps postperturbation (post1_L_–post4_R_; Fig. 3).

### Reactive locomotor responses across the eight perturbation blocks

**Figure 5.**
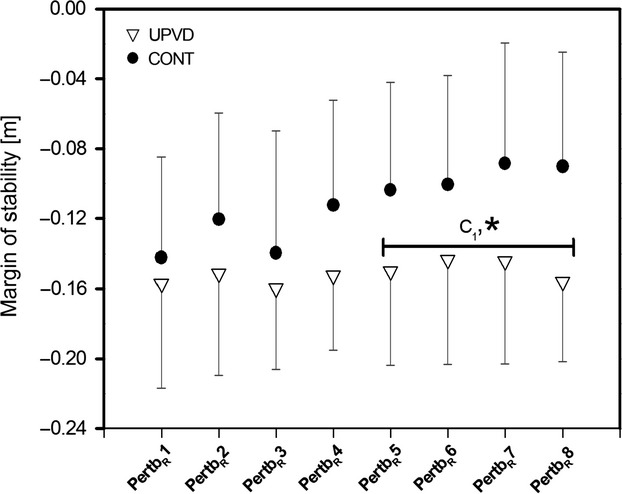
Margin of stability at touchdown of the perturbed leg (pertb_R_) for unilateral peripheral vestibular disorder (UPVD;* n *=**13) patients and matched healthy controls (CONT;* n *=**13) at the eight perturbation blocks following an unexpected perturbation during treadmill walking (set speed of 1.4 m·s^−1^; mean and SD). Negative margin of stability values indicate an unstable body configuration (i.e., subjects must make additional motor actions to preserve stability and to avoid a fall, e.g., by stepping), whereas positive margin of stability values indicate a stable body configuration (i.e., no additional motor actions are needed to preserve stability). *Statistically significant difference between the UPVD and control groups (*P* < 0.05). C_1_: Statistically significant difference to pertb_R_1 for the control group (*P* < 0.05).

The analysis of the margin of stability at touchdown of the perturbed leg in each of the eight unexpected perturbation blocks (pertb_R_ in block_1_ to pertb_R_ in block_8_) demonstrated a significant subject group x perturbation block interaction (*P* < 0.05; Fig. 5). The simple contrast test revealed no significant differences in margin of stability at touchdown of the perturbed leg between subject groups within the first four perturbation blocks (block_1_ to block_4_) but demonstrated significantly higher margin of stability values for the control subjects compared to the UPVD patients for the last four perturbation blocks (block_5_ to block_8_; *P* < 0.05; Fig. 5). Accordingly, the control subjects showed significantly (*P* < 0.05) higher margin of stability values for the last four unexpected perturbations (pertb_R_ in block_5_ to block_8_) compared to the margin of stability at the first unexpected perturbation (pertb_R_ in block_1_; see Fig. 5). For the UPVD patients, the margin of stability at touchdown of the perturbed leg for any subsequent perturbation block (pertb_R_ in block_2_ to pertb_R_ in block8) was not significantly different from the first unexpected perturbation block (pertb_R_ in block_1_; Fig. 5). Furthermore, the adaptation magnitude was significantly (*P* < 0.05) greater for the healthy control participants compared with the UPVD patients, with controls and UPVD patients achieving mean magnitudes of 25.5% (SD30.2) and 0.4% (SD25.3), respectively. There was no significant subject group effect, perturbation block effect or perturbation block x subject group interaction on base of support (range across the eight perturbation blocks and the two subject groups from 66.2 to 63.3 cm), margin of stability (7.1 to 4.9 cm) or extrapolated center of mass (59.5 to 57.5 cm) at touchdown of the contralateral leg left leg prior to perturbation. Hence, the base of support, margin of stability and extrapolated center of mass values for all preperturbation steps (pre_L_ in block_1_ to pre_L_ in block_8_) were not significantly different from baseline (nonperturbed walking) for either subject group.

## Discussion

This study aimed to examine the recovery responses (stability recovery actions) and adaptive feedback potential in dynamic stability of UPVD patients and matched healthy control subjects during perturbed treadmill walking. In order to best isolate the reactive, feedback‐driven response, the perturbations were unexpected, with no warning, the duration between onset of perturbation and touchdown was short, and the possible presence of preperturbation feedforward motor adjustments was checked. Süptitz et al. (2013) recently demonstrated that such a method may be effective in analyzing feedback‐driven locomotor corrections during perturbed gait. We hypothesized that recovery behavior would be less effective and adaptive feedback potential in dynamic stability during perturbed walking would be diminished in UPVD patients compared to matched healthy controls. It was found that, compared to matched healthy controls, UPVD patients required at least two more recovery steps to return to margin of stability baseline level after an unexpected perturbation while walking. This clearly illustrates a UPVD patient related deficit in dynamic stability control during perturbed walking. Moreover, after repeated exposure to the perturbation task, significant adaptive improvements in dynamic stability at touchdown of the perturbed leg were seen only in the healthy controls and the reactive adaptation magnitude was significantly greater for the control participants compared to the UPVD patients. Therefore, the results support the hypothesis that UPVD patients have a diminished ability to effectively cope with unexpected perturbations while walking due to a slower recovery to margin of stability baseline values following perturbations and an apparent lack of feedback‐driven locomotor adaptations.

Nonperturbed walking (baseline) revealed similar positive margin of stability values at touchdown among both subject groups (on average about 6 cm). This indicates that walking on the treadmill at 1.4 m·s^−1^ had a similar dynamic stability demand for both subject groups. One might argue that the current positive margin of stability values may contradict some previous findings (Bierbaum et al. 2010, 2011; Höhne et al. 2011) that showed negative margin of stability values at touchdown during nonperturbed walking (range of −1.7 to −11.9 cm). However, the differences are likely related to the different gait velocities of the studies (1.8–2.0 m·s^−1^ vs. 1.4 m·s^−1^ in this study) as the base of support and extrapolated center of mass at touchdown are differentially influenced by increased velocity, with the extrapolated center of mass affected to a greater extent (Süptitz et al. 2012).

After the first unexpected perturbation to the swing phase of the right leg in block_1_, margin of stability values at touchdown of the perturbed leg (pertb_R_ in block_1_) significantly decreased (*P* < 0.05; Fig. 3) for both subject groups with mean values of about −15 cm, with no significant differences between UPVD patients and controls. The appreciably negative margin of stability values demonstrates that the perturbation was appropriate to initiate an unstable body position at touchdown for the subjects. During the first step following the perturbation (post1_L_) in block_1_, both subject groups demonstrated an increased base of support at touchdown compared to at touchdown of the perturbed leg, but did not exceed the position of the extrapolated center of mass, resulting in an unstable body position (i.e., negative margin of stability) at touchdown for both groups. The controls were able to significantly increase their base of support in comparison to their baseline (i.e., nonperturbed walking) leading to higher (less negative) margin of stability values compared to the UPVD patients. By increasing the base of support more than the UPVD patients, the control subjects created a more advantageous body position at the first recovery step, positively influencing dynamic stability during the following recovery steps. A diminished ability to take a large anterior recovery step is a strong predictor for a higher fall risk (Maki and McIlroy 2006). Therefore, the above results suggest that the known increased fall risk in UPVD patients may be, in part, due to a diminished ability to effectively enlarge their base of support after an unexpected perturbation while walking.

In the course of several unexpected gait perturbations, control subjects showed significant increases in margin of stability (less negative values) at touchdown of the perturbed leg from the fifth to eighth perturbation blocks compared to the first block, attributable to a widened base of support in the eighth block. Due to the short duration between the onset of the perturbation and touchdown of the perturbed leg, and the lack of warning about the perturbation, we can suggest that the observed motor response at the perturbed leg was, to a large extent, attributed to an improvement in the reactive, feedback‐driven response. The current results confirm previous findings (Pavol et al. 2004; Bierbaum et al. 2011) that healthy adults have the potential to adapt their recovery response to perturbations in a feedback‐driven manner. We may speculate that, in this study, the internal representation of the postperturbation recovery steps and the motor response to the perturbation may have been continually updated with each perturbation, leading to a gradual improvement in recovery behavior in the healthy control subjects.

In contrast to the above findings, the examined UPVD patients showed no significant changes in margin of stability for the perturbed leg across the eight perturbation blocks, demonstrating similar values in the first and last block. Accordingly, the reactive adaptation magnitude after eight unexpected perturbations was significantly higher for the control group than the UPVD patients (25.5% vs. 0.4%). The current results, therefore, indicate that unilaterally disturbed vestibular sensory feedback information may negatively affect the accuracy of the updating of the internal representation of the internal and external mechanical environment during perturbed gait, which may result in a lack of adaptation of the reactive response to perturbations.

The finding that UPVD patients needed more steps to reach MS baseline in the final block compared to controls may support the suggestion that the integration of vestibular and somatosensory feedback is necessary for postural adjustments to some degree (Horak et al. 2001). However, the above‐described improved recovery behavior may not have been solely caused by feedback‐driven‐reactive motor adjustments, due to task experience of the perturbation aiding cerebellar controlled feedforward adjustments (Morton and Bastian 2006).

UPVD patients demonstrated an inability to increase their base of support greater than baseline level during the first step postperturbation in the first or final block. This deficient motor response resulted in a mechanically ineffective body position, negatively affecting the subsequent recovery steps, delaying the return to baseline margin of stability values for the UPVD patients. This delayed return demonstrates a persistent increased risk of falling in UPVD patients after repeated practice of the perturbation task, signifying the possible role of the vestibular system in the adaptation of the reactive response to repeated perturbations.

Analysis of the step prior to perturbation in all perturbation blocks (pre_L_ in block_1_ to block_8_) revealed no significant differences in the margin of stability, base of support, or extrapolated center of mass compared to baseline for either subject group. This suggests that there were no clear predictive adjustments in gait or posture which influenced the recovery behavior following any of the perturbations. While anxiety may have caused the recovery behavior of four subjects that grasped the treadmill handrails, this may have been due to insufficient recovery responses to the perturbation. Having excluded these participants from the statistics, our results may underestimate the impact of UPVD on dynamic stability and could have been more pronounced had they been included in the analysis.

In conclusion, our results demonstrate a deficiency in the ability of UPVD patients to effectively cope with unexpected perturbations while walking, before and after repeated practice of the perturbation task. UPVD patients showed a diminished recovery response, characterized by an inability to significantly widen their base of support following the perturbations and an apparent absence of adaptive feedback potential in dynamic stability control. This suggests that a lack of accurate vestibular sensory feedback information may result in diminished corrections and adaptations of locomotor behavior during perturbed walking, increasing the risk of falls during walking, and supports the notion that the vestibular system may be important for the adaptation of feedback‐driven‐reactive responses during locomotion.

## Acknowledgments

We thank Martin Küssel‐Feldker and Thomas Förster and their teams for their technical assistance and support throughout this research project.

## Conflict of interest

No conflicts of interest, financial or otherwise, are declared by the authors.
